# Supplementary material for: Application of the African Philosophy Ubuntu to the Care of Families With Infants Undergoing Therapeutic Hypothermia for the Treatment of Hypoxic‐Ischemic Encephalopathy
Source: Nurs Philos. 2026 Apr 26;27(2):e70086. doi: 10.1111/nup.70086 (PMC13110855; doi:10.1111/nup.70086)
Supplement: Supplementary file 1 — Supporting File [file NUP-27-e70086-s001.docx]

**Application of the African Philosophy *Ubuntu* to the Care of**

**Families with Infants Undergoing Therapeutic Hypothermia for the Treatment**

**of Hypoxic-Ischemic Encephalopathy**

**Abstract**

Neonatal intensive care units throughout the world strive to provide family-integrated care guided by the family-centered care philosophy. Under the family-integrated model of care, families are considered a member of the healthcare team and the primary caregivers to their newborn. Families experiencing intensive care related to their newborn acquiring hypoxic-ischemic encephalopathy shortly before, during or after birth, are commonly prevented from holding or participating in the care of their newborn in the first few days of life. These restrictive practices are the very antithesis of family-centered or family-integrated care. The exclusion of families in the context of family-integrated care may be a result of the dissent amongst families, healthcare providers and scholars worldwide regarding the prioritization of family-centered care philosophy. The African philosophy of *Ubuntu* shares many of the concepts of the family-centered care philosophy. This discursive paper will explore the competing frameworks and how the application of *Ubuntu* philosophy for the care of infants diagnosed with hypoxic ischemic encephalopathy lends to holistic care in the neonatal intensive care unit.

**Keywords**: Ubuntu, family-centered care, family-integrated care, neonatal hypoxic ischemic encephalopathy, therapeutic hypothermia, neonatal intensive care unit

**Application of the African Philosophy *Ubuntu* to the Care of Families with Infants Undergoing Therapeutic Hypothermia for the Treatment of Hypoxic-Ischemic Encephalopathy**

**Introduction**

The neonatal intensive care unit (NICU) provides highly specialized care to infants who experience a wide variety of health conditions including prematurity, congenital anomalies, genetic disorders, illness or birth trauma. Birth trauma may result in neonatal hypoxic-ischemic encephalopathy (HIE), a devastating neurological insult. HIE occurs because of impaired cerebral blood flow during the intrapartum or antepartum stages of labour and delivery (Beltempo et al., 2022). Globally, HIE is the second leading cause of death in the neonatal population (Cannavò et al., 2023) and survivors are at a significant risk of severe neurological injuries and poor long-term neurodevelopmental outcomes (Bäcke et al., 2022).

Therapeutic hypothermia (TH), the only available evidence-based treatment for HIE (Beltempo et al., 2022), involves lowering the core body temperature to approximately 33 degrees Celsius over a 72-hour period, using specialized equipment such as thermal mattresses (Molloy et al., 2023). Randomized controlled trials demonstrated a reduction in the risk of future neurological disability in infants diagnosed with moderate to severe HIE when treated with TH within the first 6 hours after the initial neurological insult (Azzopardi et al., 2014; Gluckman et al., 2005; Shankaran, et al., 2005; Shankaran et al., 2012). Induced hypothermia provides neuroprotection by slowing cerebral metabolism and energy utilization and subsequent brain cell death that would otherwise result in further brain injury (Cannavò et al., 2023). Newborns treated with TH require intensive care including mechanical ventilation, invasive central lines, urinary catheterization, amplitude integrated electroencephalography monitoring along with temperature probes that allow the cooling equipment to maintain the newborn’s temperature within the set parameters.

Along with the profound distress parents experience learning their infant may have a brain injury, admission to the NICU often results in separation of the parent-infant dyad. Separation is a significant source of stress and anxiety for families (Banerjee et al., 2018). Numerous studies have reported long lasting negative effects of a NICU admission on parental wellbeing (Ferrari et al., 2022) and the harmful consequences on maternal-infant bonding and attachment (Ciciolla et al., 2024). Emerging evidence has revealed parental holding during TH decreases maternal stress (Craig et al., 2019; Fox et al., 2025), decreases infant stress (Fox et al., 2025), increases maternal-infant bonding (Craig et al., 2019) and may provide pain control in infants (Gançarski et al., 2025). However, most NICUs worldwide restrict parents from holding their newborn while undergoing therapeutic hypothermia (Craig et al., 2019; Lee et al., 2024, Odd et al., 2021).

During a NICU admission, infants experience repeated exposure to stress-provoking stimuli including noise, light and a plethora of painful procedures. The cumulative exposure to noxious stimuli negatively affects infant growth and development (Kutahyalioglu & Scafide, 2023). Parental presence in the NICU provides a buffer or safeguard for infants against the negative sequelae that may result from the NICU environment (Franck & O’Brien, 2019). Research suggests that approaches designed to support parent-infant caregiving relationships, such as parental presence, can reduce disorganised attachment and mitigate the impact of disruptions in parental bonding (Ciciolla et al., 2024).

Family-centered care (FCC) and interventions such as family-integrated care (FiCare) provide a guide to support parental presence by promoting unrestricted access, encouraging parental involvement in daily care while supporting parent’s emotional well-being (Carew et al., 2024). FCC is a well-established philosophical conceptual model of care that guides and prioritizes the nursing care in the NICU by recognizing the family’s strengths, differences, and individual needs (Kuo et al., 2012). Core principles of FCC include collaboration, mutual respect, shared decision making and participation of care (Abukari & Schmollgruber, 2023). FiCare, guided by the philosophical underpinnings of FCC, extends the recognition of families by integrating them into the multidisciplinary care team (Chen & Dong, 2022; Dien et al., 2022). This ensures that families are actively involved with care decisions. However, the degrees of practice uptake of FCC and FiCare vary widely across NICUs and patient contexts.

We argue that conceptual clarity surrounding NICU care approaches grounded by *Ubuntu* philosophy has the potential to enhance acceptance amongst NICU nurses caring for families with infants receiving intensive care related to a HIE diagnosis. To situate this argument, FCC and FiCare will be defined and described in the context of the NICU. Issues surrounding the lack of conceptual clarity, confusion regarding the philosophical underpinnings and application of FCC and FiCare will be discussed. This context is intended to encourage reflection on the discourse between FCC and FiCare as seemingly ambiguous frameworks. Next, we will introduce *Ubuntu*, an African philosophy to inform holistic care of families with infants experiencing HIE and receiving TH. Given that philosophy leads to deeper understanding (Maimuna, 2022), exploring *Ubuntu* philosophy can facilitate realignment within caring frameworks in relation to the neonatal population impacted by HIE.

**Care Approaches in the Neonatal Intensive Care Unit**

**Family-Centered Care**

Recognizing the importance of family presence on the short- and long-term health outcomes of infants requiring critical care, many NICUs across the world have adopted FCC as philosophy of care (Kutahyalioglu et al., 2023). The FCC philosophy challenges the paternalistic and hierarchical traditional biomedical model by providing “a comprehensive and holistic care of patients and their families with an emphasis on family participation, respect for families’ preferences, needs, and differences, as well as transparent communication and knowledge sharing” (Lee, 2024, p.171). The principles for FCC in the NICU setting include the following core concepts: respect; diversity; strengths based; choice; flexibility; information sharing; support; collaboration and empowerment (Franck & O’Brien, 2019). During the NICU stay, families are encouraged to contribute and participate in the care of their infant in a capacity deemed appropriate by the family (Al-Motlaq et al., 2019).

Utilizing FCC as a care philosophy is considered an important element in clinical decision making to deliver high-quality patient care. Western and non-Western scholars have examined FCC using a range of methodologies across diverse settings resulting in multiple, competing conceptualizations (Al-Motlaq et al., 2024). For example, Hutchfield’s (1999) concept analysis of FCC identified two contrasting views of FCC: one that emphasizes family cooperation and respect, and another, less collaborative, that positions nursing as the “gatekeeper” in patient care. Competing terminology, fundamental misperceptions and confusion provide an added challenge (Coyne at al., 2018; Feeg et al., 2016; Moradian, 2018; Park et al., 2018). Noting the ambiguity surrounding FCC philosophy of care and variability in research focusing on FCC, Al-Motlaq et al. (2019) used a modified Delphi technique with consensus methodology to develop an international list of descriptors for FCC that would serve as the foundation for future empirical psychometric research on FCC in nursing. The authors report that ‘there is no single, cohesive vision of FCC practice to guide nurses, meaning that FCC is used in different ways in different settings based on individual beliefs and views.” (p. 463), which may explain some of the confusion surrounding implementation and poor uptake of FCC.

Neonatal nurses play a crucial role in promoting and supporting the principles of FCC (Dall’Oglio et al., 2022). The philosophical limitations of how FCC is conceptualized may lead to challenges for neonatal nurses to truly embrace FCC as a philosophy of care (Dien et al., 2022). Additional constraints faced by neonatal nurses include inadequate resources, lack of support from leadership or inadequate education on the tenets of FCC (Franck et al., 2021). A recent systematic review examining the factors affecting nurses’ ability to provide FCC noted barriers and facilitators related to the nursing profession itself, the family unit and the healthcare system (Alqarawi & Alhala, 2024). Nursing factors related to unsuccessful FCC implementation included practice background, years of experience, education, attitude and communication skill. Additional barriers included human resources, inadequate staff training, operating in a high-stress environment, time constraints and restrictions on the nurse’s authority to share information. Interestingly, none of the studies included in the systematic review examined a theoretical model to explain what factors impacted nurses practice of FCC. Kutahyalioglu et al. (2023) investigated the challenges of FCC implementation in the NICU setting. From the perspective of NICU nursing study participants, “the delivery of FCC involves respectful engagement and participation of multiple internal and external stakeholders who have committed to a mutually agreeable mission and philosophy” (p.464). This statement is particularly impactful since the history, evolution and philosophical underpinnings of FCC remain unclear (Al-Motlaq et al., 2019; Coyne et al, 2018). Moreover, FCC is inconsistently utilized, and its acceptability varies amongst healthcare providers (Coyne at al., 2011; Franck & O’Brien, 2019; Franck et al., 2024; Kutahyalioglu et al., 2023; Larocque et al., 2021). Moreover, nurses find FCC challenging to implement into practice due to confusion over how much responsibility to share with parents, internal conflict with what is required of them professionally and the perception of loss of power (Kutahyalioglu et al., 2022).

The NICU healthcare team consists of numerous disciplines who consult and collaborate with various other pediatric medical specialties in the care of the hospitalized infant. With such a diverse care team, there are many competing philosophies and ideas of what FCC means in the context of the NICU. Members of the neonatal interdisciplinary team may view FCC solely as a nursing role while simultaneously resisting strategies supporting the care philosophy including providing unlimited parental access and parental presence during interdisciplinary rounds (Boyle & Cusack, 2020). Coupled with an unclear epistemological grounding (Al-Motlaq et al., 2024), these factors contribute to the complexity of implementing and delivering FCC in the NICU (Feeg et al., 2016). Despite these conceptual and philosophical limitations, FiCare has emerged as a model of care building on the FCC philosophical framework (Chen & Dong, 2022; Dien et al., 2022).

**Family-Integrated Care**

FiCare originated from Estonia in the 1970s (Levin, 1994) based on the “conceptual model of the ‘psychological and biological umbilicus,’ which proposes that this connection binds the mother and infant together during the early weeks of life” (p. 39). FiCare is a multi-component intervention care strategy aimed to empower families in becoming the primary caregiver for their infant by integrating them into the multidisciplinary team (Patel et al., 2018). Core components involve all aspects of care (e.g. vital sign monitoring, diaper changes, nasogastric feeding, endotracheal tube suction), participation on neonatal rounds, shared decision-making regarding medical management, skin-to-skin care and more. In one study, mothers enrolled in the FiCare cohort reported the development of strong, trusting relationships with healthcare providers (Dien et al., 2022).

FiCare operates on 4 pillars: 1) Interdisciplinary staff education and support to promote family involvement in neonatal care; 2) Parent education allowing for knowledge sharing and establishment of parental confidence and skills in the care of their infant; 3) NICU environment design allowing for parental presence; 4) Psychosocial support for families (Bradford-Duarte & Gbinigie, 2022; Bueno et al., 2023). Unlike the broad philosophy of FCC, the interventional model of FiCare is well defined. Clinical trials and quality improvement initiatives demonstrate an improvement in infant and parental outcomes in units operating under the FiCare model (Banerjee et al., 2018; Benzies et al., 2020; Franck et al., 2020; Hei et al., 2021; Moreno-Sanz et al., 2021; Murphy et al., 2021; O’Brien et al., 2015; van Veenendaal et al., 2022).

FiCare can be implemented in any NICU design setting, it is best achieved when parents are provided the option to room-in 24 hours a day. However, few NICUs in Canadian hospitals and even less globally, have the structural design to offer this type of care (Public Health Agency of Canada [PHAC], 2020). The preterm infant population has been the predominant focus of research involving FiCare (Dien et al., 2022; Franck et al., 2020; Franck et al., 2022; Janvier et al., 2022; O’Brien et al., 2013). Newborns treated with TH for HIE are by policy, solely term infants or late preterm infants and are generally critically ill and clinically unstable. Though FiCare is helpful to many families, Janvier et al. (2022) identified possible ethical issues surrounding the provision of FiCare noting that if FiCare is not applied with sensitivity and awareness of the reality of each family, it may cause harm. For examples, placing expectations on families to be present in the NICU for a scheduled number of hours per day, participate in interdisciplinary rounds, make decisions and provide hands on care for infants may lead to parental distress, feelings of guilt and frustration if they are not ready or able (Janvier et al., 2022). Competing responsibilities such as job requirements, older children at home, pets and extended family may lead to an added pressure for many families. Along with the diagnosis of HIE, some families may withdraw from continued presence in the NICU (Bäcke et al., 2021). However, through supportive care practices, staff strongly influence parents comfort with continued presence in the NICU (Bäcke et al., 2021).

When reflecting on the parent-infant relationship, FiCare seems intuitive. However, it necessitates a cultural and political change within the NICU multidisciplinary team. FiCare requires nursing to shift their professional role as primary caregiver and “expert” to a supportive, mentoring role (Patel et al., 2018), that shares in the decision-making, thus relinquishing power/control associated with their role in a hierarchical healthcare system. A shift in both philosophical mindset and clinical practice (discourse) would be required to incorporate FiCare in the care of NIC families which, at the same time, remains contingent upon the acceptance of FCC as guiding care philosophy (Banerjee et al., 2018). It appears that a fundamental shift in the prevailing biomedical discourse is required. Ontological questions about who is, should be, and could be in control of primary care of infants with HIE within healthcare institutions are necessary to provide clarity.

**Caring for Families with Infants Undergoing** **Therapeutic Hypothermia**

Caring for newborns receiving TH is the very antithesis of newborn care – for both parents and nurses. In ideal circumstances, newborn care focuses on immediate uninterrupted skin-to-skin, warmth, physical touch, stimulation and establishment of feeding. The diagnosis of HIE often follows a traumatic birth and resuscitation. The chaotic and unfamiliar environment of the NICU reduces a joyful time in a family’s life to a time of distress and confusion. Parenting consists of keeping the infant comfortable within an institutional context dominated by technical equipment maintaining unnatural body temperature for their newborn. Even when NICUs have a family dedicated sleep space, most parents feel physically and emotionally separated from their newborn while the newborn is receiving TH (Bäcke et al., 2020; Nassef et al., 2012). Though there is a limited amount of available research on family’s experiences with infants admitted to the NICU with HIE, it is without question that families experience a significant amount of trauma with lasting effects upon discharge home and beyond (Heringhaus et al., 2013; Nassef et al., 2020; Sagaser et al., 2024).

Despite evidence from several small studies demonstrating that holding during the cooling process is not unsafe (Craig et al., 2019; Odd et al., 2021), it is commonplace for parents to be prevented from holding their babies until the rewarming process is complete - approximately 72 hours post TH implementation. In some NICUs, parents are discouraged from providing any physical touch to their infant while others may allow parents to be involved in diaper changing and basic care based on the comfort level of the staff (Craig et al., 2018; Sangaser et al., 2024). The lack of parental involvement in care including ability to hold, absence of skin-to-skin contact or partaking in normal newborn care such as diaper changing and feeding is detrimental to parent-infant attachment and bonding (Heringhaus et al., 2013; Lemmon et al., 2017). A recent study revealed that while all families whose newborns received TH for treatment of HIE experienced high levels of depression, parents that were able to hold their baby during TH had lower levels of depression and increased bonding scores than those parent that were not permitted to hold (Ingram et al., 2024). In a survey regarding parental perceptions of TH, 84% of parents responded that bonding was compromised by the restriction of physically touching and holding their baby (Thyagarajan et al., 2018).

Of the few available studies on parental experiences with TH, Nassef et al. (2013) noted that FCC was not fully implemented for families whose babies were undergoing TH due to separation of parents from their newborn. In a more recent qualitative descriptive study exploring the parental viewpoints and experiences of TH in NICU, where FCC was the philosophy of care, a participant stated that “even though the focus was on the care of the sick infant the staff had time to chat and ask the parents about how they and their other children were doing” (Nassef et al., 2020, p.4198). A second study participant expressed satisfaction with the overall care but expressed frustration with the inconsistent provision of FCC. Though not generalizable, these experiences showcase how FCC is often not practiced as intended when newborns are receiving treatment for HIE in the NICU.

Despite evidence that the parent-infant dyad is inextricably bound, the critically ill or unwell infant is the sole focus of care in the NICU (Ferrari et al., 2022). This model of care is contradictory to FCC and FiCare. Whereas Western philosophies have guided the creation of nursing frameworks from a positivist lens, that privileges a biomedical approach, *Ubuntu* is principally a relational philosophy focusing on community (Bayuo, 2024).The application of a philosophy such as *Ubuntu* can provide ontological (“I am because we are”) grounding to FCC/FiCare allowing the multidisciplinary NICU staff to approach care for infants with HIE from a more humanistic as opposed to biomedical lens by centering the parent-newborn-healthcare team as relationally intertwined. The core values of *Ubuntu* may strengthen supportive care and foster to greater family involvement – such as the willingness to offer holding during TH.

***Ubuntu* Philosophy**

*Ubuntu* is a traditional African philosophy originating from the Bantu people of South Africa (Chigangaidze & Chinyenze, 2022). The word *Ubuntu* exists within many spoken languages and is a guiding philosophy for individuals and communities throughout Africa (Hanks, 2008). Considered both a world view (humanistic as opposed to positivist) and a relational philosophy, people are thought to possess the qualities of Ubuntu if they demonstrate warmth, harmony, empathy and understanding (Chigangaidze & Chinyenze, 2022). *Ubuntu*, as a relational philosophy, rejects the Western philosophical stance of Cartesian dualism, in which the individual and environment are understood as separate entities (Bayuo, 2025). Instead of focusing on individualism, *Ubuntu* focuses on the importance and significance of community and the responsibility each member has to one another (Krol et al., 2024). Community and the individual are interwoven and mutually constitutive (Ewuoso & Hall, 2019) where caring is viewed as communal responsibility (Tembo, 2024). *Ubuntu* is characterized by the interconnectedness between people, families and the environment, highlighting the value of interpersonal relationships (Chigangaidze & Chinyenze, 2022).

Though difficult to articulate, define and interpret for Western societies with individualistic ideologies, Archbishop Desmond Tutu provided one of the most well-known descriptions of *Ubuntu*. In the book “*God Has a Dream*” (Tutu & Abrams, 2005), Archbishop Tutu wrote “[Ubuntu] is the essence of being human. It speaks of the fact that my humanity is caught up and is inextricably bound up in yours. I am human because I belong. It speaks about wholeness; it speaks about compassion.” (p. 22). U*buntu* is rooted in the core values of caring, closeness, collaboration, compassion, communitarianism, conformity, conscience, empathy, generosity, genuineness, hospitality, interdependence, kindness, respect, sharing, support, tolerance, unity and embodies humanness (Mulaudzi et al., 2009). However, the philosophy is not simply a collection of positive human traits; it is the very essence of living through daily expressions of love while creating a harmonious relationship with the surrounding community (Hanks, 2008). As such, *Ubuntu* is lived through daily practices with a potential to also guide family nursing practice. The significance to family nursing is in the recognition of the relationship between humans. It is the centrality of the relationship instead of individuals in isolation. If the relationship(s) is/are the focus of care, it follows that an individualistic focus is incomplete. Relationships are inherently between humans and their environment, always connecting at least two entities, or a dyad. It follows that focusing care on the family/infant *relationship* ensures, at a minimum, inclusive care.

The *Ubuntu* recognition of the connection between individual-family-environment is consistent with family-centered care approaches (Mulaudzi & Gundo, 2024). Additionally, the values of *Ubuntu* align with an ethics of care inherent in nursing culture (Mulaudzi et al., 2009), the principles of holistic nursing (Muhammad-Lawal et al., 2023) and FCC (Matshaka et al., 2024). Figure 1 depicts a conceptual framework situating FiCare within FCC grounded by *Ubuntu* as an overarching philosophical foundation. The concentric circles visually demonstrate how *Ubuntu* provides ontological and epistemological guidance to FCC as a relational approach while the FiCare model of care operationalizes the core tenets of FCC. The outer layer illustrates the foundational *Ubuntu* values (caring, closeness, compassion, conformity, conscience, empathy, generosity, genuineness, hospitality, kindness, unity and humanness) while the middle layer exhibits the overlapping shared values between FCC and *Ubuntu* (collaboration, communitarianism, interdependence, respect, sharing, support) and tenets unique to FCC (choice, empowerment, flexibility, strength-based). FiCare is nested within the concentric circles demonstrating actionable organizational changes (increased parental support, knowledge sharing), realignment of nursing care (role shift acceptance, creation of reciprocal relationships, provision of holistic care). The conceptual framework visually illustrates how Ubuntu offers a foundational reorientation of caring ethics and nursing practice.

[insert Figure 1. *Relationship between caring approaches of Ubuntu, FFC and FiCare (authors original work*).

By depicting Ubuntu as a philosophical grounding for FCC, we assert that FICare can be re-interpreted and applied in the NICU. However, academic scholars argue that an African worldview and philosophy cannot be justly or adequately described outside of Africa (Nolte & Downing, 2019). Mokgoro (1997) states:

“To define an African notion in a foreign language and from an abstract as opposed to a concrete approach is to defy the very essence of the African worldview and can also be particularly elusive ... because the African worldview cannot be neatly categorised and defined, any definition would only be a simplification of a more expansive, flexible and philosophically accommodative idea” (Mokgoro, 1997, as cited in Anofuechi & Klaasen, 2024, p.2)

Knowing this, it is important to recognize that FCC and FiCare are predominantly reflective of the Western medical model, generally operated under a paternalistic ideology. FCC has largely been studied, promoted and implemented by researchers, clinicians and institutions located in predominately White, Western countries (Hriberšek et al., 2024). The Western scientific community have been conditioned to systematically scrutinize, analyse, breakdown, fragment, categorize and reorder concepts (Müller & Trahar, 2016). *Ubuntu* “cannot be neatly categorised and defined” and “any definition would only be a simplification of a more expansive, flexible and philosophically accommodative idea” (Anofuechi & Klaasen, 2024, p.2). Surprisingly, much of the literature related to *Ubuntu* has been written and published by non-African scholars (van Breda, 2019). Western scholars must be astutely aware of oversimplifying or reframing Indigenous philosophies and reflect on how the positionality of Whiteness shapes the use of African concepts within Western theoretical frameworks to avoid epistemic injustice and silencing other understandings and contributions to knowledge (van Breda, 2019).

When applied in a healthcare setting within or outside of Africa, the *Ubuntu* philosophy emphasizes the creation of a reciprocal relationship in a supportive environment where nurses advocate for holistic care (Muhammad-Lawal et al., 2023; Mulaudzi et al., 2022). Families thrive in environments organized to support their basic needs. With respect to the NICU and families caring for babies with HIE, ensuring the family’s success in the NICU environment, institutional policies, including care philosophies need to be holistic and all-encompassing regardless of the level of acuity of the newborn.

Using participatory action research design, Mulaudzi & Gundo (2024) developed an *Ubuntu* community model of nursing. The model incorporates themes based on the value of a person, environment, values in nursing and principles of *Ubuntu*. It shares the principles of FCC and FiCare in a clear and concise manner while incorporating the philosophical tenets and core values of *Ubuntu*. Literature about families whose newborns are diagnosed with HIE and receiving care in the NICU indicates many common themes incorporated in the model including: parent’s desire in decision-making (Bäcke et al., 2021; Sagaser et al., 2024); shared responsibility in the care of their baby either through holding/touch/newborn care (Bäcke et al., 2021; Biskop et al., 2019; Pilon et al., 2021; Sagaser et al., 2024); communication, collaboration and information sharing with the healthcare team (Bäcke et al., 2021; Biskop et al., 2019; Craig, et al., 2018; Nassef, et al. 2020; Pilon et al., 2021; Sagaser et al., 2024; Thyagarajan et al., 2018); developing a sense of belonging (Biskop et al., 2019; Nassef, et al., 2020); and empowerment (Heringhaus et al., 2013; Lemmon et al., 2017; Nassef et al., 2013; Pilon et al., 2021; Sagaser et al., 2024). Applying the *Ubuntu model of nursing* (Mulaudzi & Gundo, 2024, p.5) to holistic nursing care practices in the NICU may address many of the concerns expressed by families and nurses regarding FCC/FiCare including the roles, responsibilities and expectations of both family and nursing. It may support existing NICU approaches to care by providing philosophical clarity to the entire NICU team.

Actualizing *Ubuntu* in the context of providing FCC and FiCare in the NICU may redefine the concept of community and accentuate the responsibility to something much greater than the NICU team – essentially the family as a core unit embedded in the NICU community. When authentically applied in the NICU setting, specifically for families with newborns receiving TH, *Ubuntu* core values can be operationalized through clinical practice. For example, the core concepts of Ubuntu can be demonstrated in the NICU by providing family specific care for newborns with HIE including acknowledging the unique, stressful experience related to TH (*caring/empathy*); providing compassionate honest information related to clinical condition and potential life altering outcomes related to HIE (*compassion/conscience/sharing*); ensuring open communication and dialogue (*respect*); involving families in all aspects of clinical care and decision-making (*communitarianism/unity*) and encouraging families to provide hands on care to their newborn (*closeness/hospitality*). *Ubuntu* grounds FCC by providing a solid onto-epistemological position (I am because you are). Situating FCC within *Ubuntu* may provide a philosophical lens to FiCare and assist in the clarification of this model of care and the provision of holistic, inclusive NICU care. In doing so, this can redirect how care is delivered by moving away from prioritizing clinical tasks for the infant in isolation to incorporating *Ubuntu* principles in nursing culture.

In *Ubuntu*, caring is a communal role (Chisale, 2018). Personhood is embodied through relationships within the community. In contrast, FCC prioritizes the individual (infant), with the community (family) as the relational support system fostering care practices resulting in independence. By placing the family at the center of care surrounded by community of the NICU, nursing fulfills the *Ubuntu* axiom *umuntu ngumuntu ngabantu* meaning “a person is a person through other persons” (Tembo, 2024) where a person truly becomes a person through self-determination and sense of responsibility in the service of others fostering the interconnectedness of the community. *Ubuntu*, as a relational philosophy, may reorientate NICU nurse’s positionality away from the individualistic biomedical ontology of FCC/FiCare to one where personhood encompasses relationships built through community, and care is one of reciprocal growth fostered by interdependence. If the community thrives, the individual thrives. If the individual thrives, the community thrives (Udah et al., 2025). As such, the focus of care does not privilege the biomedical needs of the infant in isolation but recognizes the biopsychosocial reality of the family unit.

**Conclusion**

Research related to family experiences with babies who require TH for treatment of HIE acquired at birth is limited. What is known reveals the exclusion of families in the care of their newborn while cooling therapy is ongoing and beyond. Holistic care approaches such as family-centered care and family-integrated care embedded in NICUs across the world, face challenges related to competing terminology, fundamental misperceptions and confusion making full implementation difficult. The values and ethos of the traditional African philosophy *Ubuntu* echo many of the principles purported by the family-centered care philosophy. *Ubuntus*’s humanistic tenets are important elements of caring found in the nursing profession (Tembo, 2024). When adopted as overarching philosophy, *Ubuntu* can provide the necessary grounding of care approaches for families in the NICU, thus facilitating the FCC approach and FiCare intervention model and empowering the healthcare team to provide the best possible care to families who experience the journey of neonatal HIE.

**References**

Abukari, A. S., & Schmollgruber, S. (2023). Concepts of family-centered care at the neonatal and paediatric intensive care unit: A scoping review. *Journal of Pediatric Nursing*, *71*, e1–e10. <https://doi.org/10.1016/j.pedn.2023.04.005>

Al-Motlaq, M. A., Carter, B., Neill, S., Hallstrom, I. K., Foster, M., Coyne, I., Arabiat, D., Darbyshire, P., Feeg, V. D., & Shields, L. (2019). Toward developing consensus on family-centred care: An international descriptive study and discussion. *Journal of Child Health Care*, *23*(3), 458–467. <https://doi.org/10.1177/1367493518795341>

Al-Motlaq, M., Foster, M., Zgambo, M., & Neill, S. (2024). Assessing the maturity of the “Family Centered Care” concept: A review of concept analyses studies. *Journal of Pediatric Nursing*, *79*, 150–156. <https://doi.org/10.1016/j.pedn.2024.09.004>

Anofuechi, B. O., & Klaasen, J. S. (2024). A critical analysis of ubuntu as the nexus of identity development in present-day Africa. *HTS Teologiese Studies / Theological Studies*, *80*(1). <https://doi.org/10.4102/hts.v80i1.8507>

Alqarawi, N., & Alhalal, E. (2024). Factors affecting family-centered care practice by nurses: A systematic review. *Journal of Pediatric Nursing*, *78*, 158–171. <https://doi.org/10.1016/j.pedn.2024.06.008>

Azzopardi, D., Strohm, B., Marlow, N., Brocklehurst, P., Deierl, A., Eddama, O., Goodwin, J., Halliday, H. L., Juszczak, E., Kapellou, O., Levene, M., Linsell, L., Omar, O., Thoresen, M., Tusor, N., Whitelaw, A., & Edwards, A. D. (2014). Effects of Hypothermia for Perinatal Asphyxia on Childhood Outcomes. *New England Journal of Medicine*, *371*(2), 140–149. <https://doi.org/10.1056/NEJMoa1315788>

Bäcke, P., Hjelte, B., Hellström Westas, L., Ågren, J., & Thernström Blomqvist, Y. (2021). When all I wanted was to hold my baby—The experiences of parents of infants who received therapeutic hypothermia. *Acta Paediatrica*, *110*(2), 480–486. <https://doi.org/10.1111/apa.15431>

Banerjee, J., Aloysius, A., Platonos, K., & Deierl, A. (2018). Family centred care and family delivered care – What are we talking about? *Journal of Neonatal Nursing*, *24*(1), 8–12. <https://doi.org/10.1016/j.jnn.2017.11.004>

Bayuo, J. (2024). African philosophy and nursing: A potential twain that shall meet? *Nursing Philosophy*, *25*(1), e12472. <https://doi.org/10.1111/nup.12472>

Bayuo, J. (2025). Indigenous African Philosophy as a Paradigm for Health and Social Care Research: A Philosophical and Methodological Discussion. *Nursing Inquiry*, *32*(2), e70002. <https://doi.org/10.1111/nin.70002>

Beltempo, M., Wintermark, P., Mohammad, K., Jabbour, E., Afifi, J., Shivananda, S., Louis, D., Redpath, S., Lee, K. S., Fajardo, C., Shah, P. S., & Canadian Neonatal Network Investigators (2022). Variations in practices and outcomes of neonates with hypoxic ischemic encephalopathy treated with therapeutic hypothermia across tertiary NICUs in Canada. *Journal of perinatology: official journal of the California Perinatal Association*, *42*(7), 898 - 906. https://doi.org/10.1038/s41372-022-01412-7

Benzies, K. M., Aziz, K., Shah, V., Faris, P., Isaranuwatchai, W., Scotland, J., Larocque, J., Mrklas, K. J., Naugler, C., Stelfox, H. T., Chari, R., Soraisham, A. S., Akierman, A. R., Phillipos, E., Amin, H., Hoch, J. S., Zanoni, P., Kurilova, J., Lodha, A., & Alberta FICare Level II NICU Study Team (2020). Effectiveness of Alberta Family Integrated Care on infant length of stay in level II neonatal intensive care units: a cluster randomized controlled trial. *BMC pediatrics*, *20*(1), 535. <https://doi.org/10.1186/s12887-020-02438-6>

Biskop, E., Paulsdotter, T., Hellström Westas, L., Ågren, J., & Blomqvist, Y. T. (2019). Parental participation during therapeutic hypothermia for neonatal hypoxic-ischemic encephalopathy. *Sexual & Reproductive Healthcare: Official Journal of the Swedish Association of Midwives*, *20*, 77–80. <https://doi.org/10.1016/j.srhc.2019.03.004>

Bradford-Duarte, R., & Gbinigie, H. (2020). Neonatal family integrated care: Ensuring a positive parental experience. *Journal of Neonatal Nursing*, *26*(5), 284–290. <https://doi.org/10.1016/j.jnn.2020.03.003>

Bueno, M., Vieira, A. C. G., & Bacchini, F. (2023). Family integrated care: New perspectives for neonatal care. *Online Brazilian Journal of Nursing*, *22*. <https://doi.org/10.17665/1676-4285.20236634>

Cannavò, L., Perrone, S., & Gitto, E. (2023). Brain-Oriented Strategies for Neuroprotection of

Asphyxiated Newborns in the First Hours of Life. *Pediatric neurology*, *143*, 44–49.

Carew, M., Redley, B., & Bloomer, M. J. (2024). Competing Tensions: Nurse Perceptions of Family-Centered Care and Parents’ Needs in Neonatal Care. *Advances in Neonatal Care*. <https://doi.org/10.1097/ANC.0000000000001136>

Chen, H., & Dong, L. (2022). The effect of family integrated care on the prognosis of premature infants. *BMC Pediatrics*, *22*(1), 668. <https://doi.org/10.1186/s12887-022-03733-0>

Chigangaidze, R., K., & Chinyenze, P. (2022). What it means to say, ‘ <i>a person is a person through other persons’</i>: Ubuntu through humanistic-existential lenses of transactional analysis. *Journal of Religion & Spirituality in Social Work: Social Thought*, *41*(3), 280–295. <https://doi.org/10.1080/15426432.2022.2039341>

Ciciolla, L., Shreffler, K. M., Quigley, A. N., Price, J. R., & Gold, K. P. (2024). The Protective Role of Maternal-Fetal Bonding for Postpartum Bonding Following a NICU Admission. *Maternal and Child Health Journal*, *28*(1), 11–18. <https://doi.org/10.1007/s10995-023-03873-4>

Coyne, I., Holmström, I., & Söderbäck, M. (2018). Centeredness in Healthcare: A Concept Synthesis of Family-centered Care, Person-centered Care and Child-centered Care. *Journal of Pediatric Nursing*, *42*, 45–56. <https://doi.org/10.1016/j.pedn.2018.07.001>

Coyne, I., O’Neill, C., Murphy, M., Costello, T., & O’Shea, R. (2011). What does family-centred care mean to nurses and how do they think it could be enhanced in practice: Family-centred care. *Journal of Advanced Nursing*, *67*(12), 2561–2573. <https://doi.org/10.1111/j.1365-2648.2011.05768.x>

Craig, A., Deerwester, K., Fox, L., Jacobs, J., & Evans, S. (2019). Maternal holding during therapeutic hypothermia for infants with neonatal encephalopathy is feasible. *Acta Paediatrica*, *108*(9), 1597–1602. <https://doi.org/10.1111/apa.14743>

Dall’Oglio, I., Mascolo, R., Portanova, A., Ragni, A., Amadio, P., Fiori, M., Tofani, M., Gawronski, O., Piga, S., Rocco, G., Tiozzo, E., Latour, J. M., & on behalf of the FCC Italian NICUs Study Group. (2022). Staff Perceptions of Family-Centered Care in Italian Neonatal Intensive Care Units: A Multicenter Cross-Sectional Study. *Children*, *9*(9), 1401. <https://doi.org/10.3390/children9091401>

Dien, R., Benzies, K. M., Zanoni, P., & Kurilova, J. (2022). Alberta Family Integrated Care^TM^ and Standard Care: A Qualitative Study of Mothers’ Experiences of their Journeying to Home from the Neonatal Intensive Care Unit. *Global Qualitative Nursing Research*, *9*, 23333936221097113. <https://doi.org/10.1177/23333936221097113>

Downing, C., & Hastings-Tolsma, M. (2016). An integrative review of Albertina Sisulu and ubuntu: Relevance to caring and nursing. *Health SA Gesondheid*, *21*, 214–227. <https://doi.org/10.4102/hsag.v21i0.956>

Ewuoso, C., & Hall, S. (2019). Core aspects of ubuntu: A systematic review. *South African Journal of Bioethics and Law*, *12*(2), 93. <https://doi.org/10.7196/SAJBL.2019.v12i2.679>

Feeg, V. D., Paraszczuk, A. M., Çavuşoğlu, H., Shields, L., Pars, H., & Al Mamun, A. (2016). How is Family Centered Care Perceived by Healthcare Providers from Different Countries? An International Comparison Study. *Journal of Pediatric Nursing*, *31*(3), 267–276. <https://doi.org/10.1016/j.pedn.2015.11.007>

Ferrari, R. M., McClain, E. K., Tucker, C., Charles, N., Verbiest, S., Lewis, V., Bryant, K., & Stuebe, A. M. (2022). Postpartum Health Experiences of Women with Newborns in Intensive Care: The Desire to Be by the Infant Bedside as a Driver of Postpartum Health. *Journal of Midwifery & Women’s Health*, *67*(1), 114–125. <https://doi.org/10.1111/jmwh.13330>

Fox, L., Cutler, A., Kaneko-Tarui, T., Deerwester, K., Evans, S., Maron, J., & Craig, A. (2025). A Pilot Randomized Control Trial of Holding During Hypothermia and Effects on Maternal and Infant Salivary Cortisol Levels. *Advances in Neonatal Care*. <https://doi.org/10.1097/ANC.0000000000001239>

Franck, L. S., Cormier, D. M., Hutchison, J., Moore, D., Bisgaard, R., Gay, C., Ngo, S., Kriz, R. M., Lin, C., Ekno, M., Ribero, D., & Sun, Y. (2021). A Multisite Survey of NICU Healthcare Professionals’ Perceptions About Family-Centered Care. *Advances in Neonatal Care*, *21*(3), 205–213. <https://doi.org/10.1097/ANC.0000000000000805>

Franck, L. S., Gay, C. L., Hoffmann, T. J., Kriz, R. M., Bisgaard, R., Cormier, D. M., Joe, P., Lothe, B., & Sun, Y. (2022). Neonatal outcomes from a quasi-experimental clinical trial of Family Integrated Care versus Family-Centered Care for preterm infants in U.S. NICUs. *BMC Pediatrics*, *22*(1), 674. <https://doi.org/10.1186/s12887-022-03732-1>

Franck, L. S., Gay, C. L., Hoffmann, T. J., Kriz, R. M., Bisgaard, R., Cormier, D. M., Joe, P., Lothe, B., & Sun, Y. (2023). Maternal mental health after infant discharge: A quasi-experimental clinical trial of family integrated care versus family-centered care for preterm infants in U.S. NICUs. *BMC Pediatrics*, *23*(1), 396. <https://doi.org/10.1186/s12887-023-04211-x>

Franck, L. S., Kriz, R. M., Bisgaard, R., Cormier, D. M., Joe, P., Miller, P. S., Kim, J. H., Lin, C., & Sun, Y. (2019). Comparison of family centered care with family integrated care and mobile technology (mFICare) on preterm infant and family outcomes: A multi-site quasi-experimental clinical trial protocol. *BMC Pediatrics*, *19*(1), 469. <https://doi.org/10.1186/s12887-019-1838-3>

Franck, L. S., Magaña, J., Bisgaard, R., Lothe, B., Sun, Y., & Morton, C. H. (2024). Mobile-enhanced Family Integrated Care for preterm infants: A qualitative study of parents’ views. *PEC Innovation*, *4*, 100284. <https://doi.org/10.1016/j.pecinn.2024.100284>

Franck, L. S., & O’Brien, K. (2019). The evolution of family‐centered care: From supporting parent‐delivered interventions to a model of family integrated care. *Birth Defects Research*, *111*(15), 1044–1059. <https://doi.org/10.1002/bdr2.1521>

Franck, L. S., Waddington, C., & O’Brien, K. (2020). Family Integrated Care for Preterm Infants. *Critical Care Nursing Clinics of North America*, *32*(2), 149–165. <https://doi.org/10.1016/j.cnc.2020.01.001>

Gançarski, L., Langlet-Muteau, C., Rondel, J., Escande, B., Koenig-Zores, C., & Kuhn, P. (2025). Physiological and behavioral stability of newborns on therapeutic hypothermia for hypoxic-ischemic encephalopathy during parental holding. *Pediatric Research*. <https://doi.org/10.1038/s41390-025-03812-9>

Gluckman, P. D., Wyatt, J. S., Azzopardi, D., Ballard, R., Edwards, A. D., Ferriero, D. M., Polin, R. A., Robertson, C. M., Thoresen, M., Whitelaw, A., & Gunn, A. J. (2005). Selective head cooling with mild systemic hypothermia after neonatal encephalopathy: Multicentre randomised trial. *The Lancet*, *365*(9460), 663–670. <https://doi.org/10.1016/S0140-6736(05)17946-X>

Gómez-Cantarino, S., García-Valdivieso, I., Moncunill-Martínez, E., Yáñez-Araque, B., & Ugarte Gurrutxaga, M. I. (2020). Developing a Family-Centered Care Model in the Neonatal Intensive Care Unit (NICU): A New Vision to Manage Healthcare. *International Journal of Environmental Research and Public Health*, *17*(19), 7197. <https://doi.org/10.3390/ijerph17197197>

Hanks, T. L. (2008). The Ubuntu Paradigm: Psychology’s Next Force? *Journal of Humanistic Psychology*, *48*(1), 116–135. <https://doi.org/10.1177/0022167807303004>

Hei, M., Gao, X., Li, Y., Gao, X., Li, Z., Xia, S., Zhang, Q., Han, S., Gao, H., Nong, S., Zhang, A., Li, J., Wang, Y., Ye, X. Y., & Lee, S. K. (2021). Family Integrated Care for Preterm Infants in China: A Cluster Randomized Controlled Trial. *The Journal of Pediatrics*, *228*, 36-43.e2. <https://doi.org/10.1016/j.jpeds.2020.09.006>

Hriberšek, M., Eibensteiner, F., Bukowski, N., Yeung, A. W. K., Atanasov, A. G., & Schaden, E. (2024). Research areas and trends in family-centered care in the 21st century: A bibliometric review. *Frontiers in Medicine*, *11*, 1401577. <https://doi.org/10.3389/fmed.2024.1401577>

Hutchfield, K. (1999). Family-centred care: A concept analysis. *Journal of Advanced Nursing*, *29*(5), 1178–1187.

Janvier, A., Asaad, M.-A., Reichherzer, M., Cantin, C., Sureau, M., Prince, J., Luu, T. M., & Barrington, K. J. (2022). The ethics of family integrated care in the NICU: Improving care for families without causing harm. *Seminars in Perinatology*, *46*(3), 151528. <https://doi.org/10.1016/j.semperi.2021.151528>

Jordan, E., Grant, M., Bhakthavalsala, S., & Chakkarapani, E. (2018). Survey of sedation, respiratory support and parental contact practices during therapeutic hypothermia for hypoxic-ischaemic encephalopathy. *Neonatal Society*. 2018, 21.

Krol, P., Einboden, R., Wong, H., Geia, L., & Tembo, A. (2024). Reimagining a nursing ecosystem in an uncertain world. *Nursing Philosophy*, *25*(4), e12501. <https://doi.org/10.1111/nup.12501>

Kuo, D. Z., Houtrow, A. J., Arango, P., Kuhlthau, K. A., Simmons, J. M., & Neff, J. M. (2012). Family-Centered Care: Current Applications and Future Directions in Pediatric Health Care. *Maternal and Child Health Journal*, *16*(2), 297–305. <https://doi.org/10.1007/s10995-011-0751-7>

Kutahyalioglu, N. S., Mallinson, R. K., Scafide, K. N., & D’Agata, A. L. (2023). “It Takes a Village” to Implement Family-Centered Care in the Neonatal Intensive Care Unit. *Advances in Neonatal Care*. <https://doi.org/10.1097/ANC.0000000000001091>

Kutahyalioglu, N. S., & Scafide, K. N. (2023). Effects of family-centered care on bonding: A systematic review. *Journal of Child Health Care*, *27*(4), 721–737. <https://doi.org/10.1177/13674935221085799>

Kutahyalioglu, N. S., Scafide, K. N., Mallinson, K. R., & D’Agata, A. L. (2022). Implementation and Practice Barriers of Family-Centered Care Encountered by Neonatal Nurses. *Advances in Neonatal Care*, *22*(5), 432–443. <https://doi.org/10.1097/ANC.0000000000000948>

Larocque, C., Peterson, W. E., Squires, J. E., Mason-Ward, M., Mayhew, K., & Harrison, D. (2021). Family-centred care in the Neonatal Intensive Care Unit: A concept analysis and literature review. *Journal of Neonatal Nursing*, *27*(6), 402–411. <https://doi.org/10.1016/j.jnn.2021.06.014>

Lee, J. (2024). Neonatal family-centered care: Evidence and practice models. *Clinical and Experimental Pediatrics*, *67*(4), 171–177. <https://doi.org/10.3345/cep.2023.00367>

Lee, K.-S., Massaro, A., Wintermark, P., Soul, J., Natarajan, G., Dizon, M. L. V., Mietzsch, U., Mohammad, K., Wu, T.-W., Chandel, A., Shenberger, J., DiGeronimo, R., Peeples, E. S., Hamrick, S., Cardona, V. Q., & Rao, R. (2024). Practice Variations for Therapeutic Hypothermia in Neonates with Hypoxic-ischemic Encephalopathy: An International Survey. *The Journal of Pediatrics*, *274*, 114181. <https://doi.org/10.1016/j.jpeds.2024.114181>

Levin, A. (1994). The Mother‐Infant Unit at Tallinn Children’s Hospital, Estonia: A Truly Baby‐Friendly Unit. *Birth*, *21*(1), 39–44. <https://doi.org/10.1111/j.1523-536X.1994.tb00914.x>

Maimuna, S. (2022). *MODEL FAMILY CENTERED CARE IN CHILDREN WITH DIABETES MELITUS: A PHILOSOPHICAL PERSPECTIVE*. <https://doi.org/10.1101/2022.07.24.22277979>

Moradian, S. T. (2018). Family-Centered Care: An Evolutionary Concept Analysis. *International Journal of Medical Reviews*, *5*(2), 82–86. <https://doi.org/10.29252/IJMR-050207>

Moreno‐Sanz, B., Alferink, M. T., O’Brien, K., & Franck, L. S. (2024). Family integrated care: State of art and future perspectives. *Acta Paediatrica*, apa.17272. <https://doi.org/10.1111/apa.17272>

Molloy, E. J., El-Dib, M., Juul, S. E., Benders, M., Gonzalez, F., Bearer, C., Wu, Y. W., Robertson, N. J., Hurley, T., Branagan, A., Michael Cotten, C., Tan, S., Laptook, A., Austin, T., Mohammad, K., Rogers, E., Luyt, K., Bonifacio, S., Soul, J. S., … McCaul, M. C. (2023). Neuroprotective therapies in the NICU in term infants: Present and future. *Pediatric Research*, *93*(7), 1819–1827. <https://doi.org/10.1038/s41390-022-02295-2>

Moreno-Sanz, B., Montes, M. T., Antón, M., Serrada, M. T., Cabrera, M., & Pellicer, A. (2021). Scaling Up the Family Integrated Care Model in a Level IIIC Neonatal Intensive Care Unit: A Systematic Approach to the Methods and Effort Taken for Implementation. *Frontiers in* Pediatrics, 9, 682097. <https://doi.org/10.3389/fped.2021.682097>

Mugumbate, J. & Chereni, A. (2019). Using African ubuntu theory in social work with children in Zimbabwe. African Journal of Social Work, 9(1), 27-34.

Muhammad-Lawal, A. T., Anokwuru, R. A., Bhana-Pemu, V., & Mulaudzi, F. M. (2023). Interrelatedness of African Care Concept of *Ubuntu* and Caring in Nursing: The Perceptions of Student-Nurses. *International Journal for Human Caring*, *27*(1), 57–67. <https://doi.org/10.20467/IJHC-2022-0005>

Mulaudzi, F. M., & Gundo, R. (2024). The views of nurses and healthcare users on the development of Ubuntu community model in nursing in selected provinces in South Africa: A participatory action research. *Nursing Outlook*, *72*(6), 102269. <https://doi.org/10.1016/j.outlook.2024.102269>

Mulaudzi, F. M., Anokwuru, R. A., Du-Plessis, M. A. R., & Lebese, R. T. (2022). Reflections on the Concomitants of the Restrictive Visitation Policy During the COVID-19 Pandemic: An Ubuntu Perspective. *Frontiers in Sociology*, *6*, 769199. <https://doi.org/10.3389/fsoc.2021.769199>

Mulaudzi, F. M., Libster, M. M., & Phiri, S. (2009). Suggestions for Creating a Welcoming Nursing Community: Ubuntu, Cultural Diplomacy, and Mentoring. *International Journal of Human Caring*, *13*(2), 45–51. <https://doi.org/10.20467/1091-5710.13.2.45>

Müller, J., & Trahar, S. (2016). Facing our whiteness in doing Ubuntu research. Finding spatial justice for the researcher. *HTS Teologiese Studies / Theological Studies*, *72*(1), 7 pages. <https://doi.org/10.4102/hts.v72i1.3510>

Murphy, M., Shah, V., & Benzies, K. (2021). Effectiveness of Alberta Family-Integrated Care on Neonatal Outcomes: A Cluster Randomized Controlled Trial. *Journal of Clinical Medicine*, *10*(24), 5871. <https://doi.org/10.3390/jcm10245871>

Nassef, S. K., Blennow, M., & Jirwe, M. (2020). Parental viewpoints and experiences of therapeutic hypothermia in a neonatal intensive care unit implemented with Family-Centred Care. *Journal of Clinical Nursing*, *29*(21–22), 4194–4202. <https://doi.org/10.1111/jocn.15448>

Ncube, L. B. (2010). Ubuntu: A transformative leadership philosophy. *Journal of Leadership Studies*, *4*(3), 77–82. <https://doi.org/10.1002/jls.20182>

Nolte, A., & Downing, C. (2019). Ubuntu—the Essence of Caring and Being: A Concept Analysis. *Holistic Nursing Practice*, *33*(1), 9–16. <https://doi.org/10.1097/HNP.0000000000000302>

O’Brien, K., Bracht, M., Macdonell, K., McBride, T., Robson, K., O’Leary, L., Christie, K., Galarza, M., Dicky, T., Levin, A., & Lee, S. K. (2013). A pilot cohort analytic study of Family Integrated Care in a Canadian neonatal intensive care unit. *BMC Pregnancy and Childbirth*, *13*(Suppl 1), S12. <https://doi.org/10.1186/1471-2393-13-S1-S12>

O’Brien, K., Bracht, M., Robson, K., Ye, X. Y., Mirea, L., Cruz, M., Ng, E., Monterrosa, L., Soraisham, A., Alvaro, R., Narvey, M., Da Silva, O., Lui, K., Tarnow-Mordi, W., & Lee, S. K. (2015). Evaluation of the Family Integrated Care model of neonatal intensive care: A cluster randomized controlled trial in Canada and Australia. *BMC Pediatrics*, *15*(1), 210. <https://doi.org/10.1186/s12887-015-0527-0>

Odd, D., Okano, S., Ingram, J., Blair, P. S., Billietop, A., Fleming, P. J., Thoresen, M., & Chakkarapani, E. (2021). Physiological responses to cuddling babies with hypoxic-ischaemic encephalopathy during therapeutic hypothermia: An observational study. *BMJ Paediatrics Open*, *5*(1), e001280. <https://doi.org/10.1136/bmjpo-2021-001280>

Park, M., Giap, T.-T.-T., Lee, M., Jeong, H., Jeong, M., & Go, Y. (2018). Patient- and family-centered care interventions for improving the quality of health care: A review of systematic reviews. *International Journal of Nursing Studies*, *87*, 69–83. <https://doi.org/10.1016/j.ijnurstu.2018.07.006>

Patel, N., Ballantyne, A., Bowker, G., Weightman, J., & Weightman, S. (2018). Family Integrated Care: Changing the culture in the neonatal unit. *Archives of Disease in Childhood*, *103*(5), 415–419. <https://doi.org/10.1136/archdischild-2017-313282>

Pilon, B., Craig, A. K., Lemmon, M. E., Goeller, A., & Newborn Brain Society Guidelines and Publications Committee. (2021). Supporting families in their child’s journey with neonatal encephalopathy and therapeutic hypothermia. *Seminars in Fetal & Neonatal Medicine*, *26*(5), 101278. <https://doi.org/10.1016/j.siny.2021.101278>

Public Health Agency of Canada, 2020. Chapter 5: Postpartum Care. In: Public Health Agency of Canada (Ed.) Family-Centred Maternity and Newborn Care National Guidelines. https://www.canada.ca/content/dam/hc-sc/documents/services/ publications/healthy-living/maternity-newborn-care-guidelines-chapter-5/mate rnity-guidelines-chapter-5-en.pdf.

Sambala, E. Z., Cooper, S., & Manderson, L. (2020). Ubuntu as a Framework for Ethical Decision Making in Africa: Responding to Epidemics. *Ethics & Behavior*, *30*(1), 1–13. <https://doi.org/10.1080/10508422.2019.1583565>

Shankaran, S., Laptook, A. R., Ehrenkranz, R. A., Tyson, J. E., McDonald, S. A., Donovan, E. F., Fanaroff, A. A., Poole, W. K., Wright, L. L., Higgins, R. D., Finer, N. N., Carlo, W. A., Duara, S., Oh, W., Cotten, C. M., Stevenson, D. K., Stoll, B. J., Lemons, J. A., Guillet, R., & Jobe, A. H. (2005). Whole-Body Hypothermia for Neonates with Hypoxic–Ischemic Encephalopathy. *New England Journal of Medicine*, *353*(15), 1574–1584. <https://doi.org/10.1056/NEJMcps050929>

Shankaran, S., Pappas, A., McDonald, S. A., Vohr, B. R., Hintz, S. R., Yolton, K., Gustafson, K. E., Leach, T. M., Green, C., Bara, R., Huitema, C. M. P., Ehrenkranz, R. A., Tyson, J. E., Das, A., Hammond, J., Peralta-Carcelen, M., Evans, P. W., Heyne, R. J., Wilson-Costello, D. E., … Higgins, R. D. (2012). Childhood Outcomes after Hypothermia for Neonatal Encephalopathy. *New England Journal of Medicine*, *366*(22), 2085–2092. <https://doi.org/10.1056/NEJMoa1112066>

Tembo, A. C. (2024). The place of philosophy in nursing. *Nursing Philosophy*, *25*(1), e12473. <https://doi.org/10.1111/nup.12473>

Thyagarajan, B., Baral, V., Gunda, R., Hart, D., Leppard, L., & Vollmer, B. (2018). Parental perceptions of hypothermia treatment for neonatal hypoxic-ischaemic encephalopathy. *The Journal of Maternal-Fetal & Neonatal Medicine*, *31*(19), 2527–2533. <https://doi.org/10.1080/14767058.2017.1346074>

Tutu, D., & Abrams, D. (2005). God has a dream : a vision of hope for our time (1st Image Books ed.). Doubleday.

Udah, H., Tusasiirwe, S., Mugumbate, R., & Gatwiri, K. (2025). Ubuntu philosophy, values, and principles: An opportunity to do social work differently. *Journal of Social Work*, *25*(4), 433–451. <https://doi.org/10.1177/14680173241312749>

Van Breda, A. (2019). Developing the notion of ubuntu as African theory for social work practice. *Social Work*, *55*(4). <https://doi.org/10.15270/55-4-762>

Van Veenendaal, N. R., Van Der Schoor, S. R. D., Broekman, B. F. P., De Groof, F., Van Laerhoven, H., Van Den Heuvel, M. E. N., Rijnhart, J. J. M., Van Goudoever, J. H. B., & Van Kempen, A. A. M. W. (2022). Association of a Family Integrated Care Model with Paternal Mental Health Outcomes During Neonatal Hospitalization. *JAMA Network Open*, *5*(1), e2144720. <https://doi.org/10.1001/jamanetworkopen.2021.44720>
